# Reply to the Cosmos on Trade Journalism

**Published:** 1889-11

**Authors:** 


### Editorial.

### REPLY TO THE COSMOS ON TRADE JOURNALISM.

THE undignified and personal editorial in the October *Cosmos* gave us as much surprise as regret, for the question had been discussed in the article on Trade Journalism from an entirely impersonal point of view, and the course of the *Cosmos* for the past few years was cited simply to strengthen our position. If the comparison had been favorable to that journal, nothing further would, probably, have been heard from it; but because it was not, the editor saw fit to reply in a manner at once discourteous and unreasonable.

The course of a journal is open for inspection, comment, and criticism, if the general good seems to require it. We deprecate personal controversies, and do not propose, in meeting ordinary questions, to enter into that kind of argument; nevertheless, we felt called upon to notice the evident dereliction of duty upon the part of the *Cosmos*, and would respectfully remind the editor that, in discussing any question pertaining to the good of the profession, we are strictly attending to our legitimate duties.

The INTERNATIONAL DENTAL JOURNAL represents a medium of expression which is entirely untrammelled by collateral business interest, and therefore represents a condition of independence that a trade journal necessarily cannot assume. This point was virtually admitted by the *Cosmos* in that no attempt was made to answer this argument. It exhibited a want of interest in the profession when the editor remarked, "The *Dental Cosmos* is not the organ of the Dental Protective Association, nor is it the organ of any society or association." That was precisely what we claimed; and if the paragraph had gone farther and said that it is, however, very watchful of the interests of its company publisher, and is essentially *its organ*, then the whole ground would have been covered, and we would not have been put to the trouble of further pursuing the subject.

Not only was the editorial undignified in character, but it was also unfair when it tried, by misrepresentation, to undervalue the service this journal has rendered the Dental Protective Association. It says:

> "What will be thought of the fact that the only two circulars authorized by the directors of the association, of which we have knowledge, have never appeared in the reading-pages of the *International Journal*, although they each had two insertions in its advertising columns?"

Now, the fact is that the circulars referred to were inserted in our advertising columns with the full knowledge and consent of Dr. Crouse, for the reason that it was proposed to keep them as standing matter; this we did, and at the same time published much information regarding the association in our editorial and reading columns.

Again we quote, italics ours:

> "It [the *Cosmos*] has given its readers *all the information needed concerning the Dental Protective Association*, and the editorial on that subject, as it appears in the current number, was in type before the issue of the journal whose uncalled-for remarks we have quoted."

From which it appears that the editor of the *Cosmos* constitutes himself the judge as to how much information is proper for the profession to receive on such matters. This is not the first time, that he has asserted a judicial prerogative on questions of general interest. The rebuke to the members of the Odontological Society of New York is still fresh in the memory of those gentlemen, and a reference to it here may not be out of place. The article may be found in the June number of the current year. In substance, it says that Dr. Merriam, in order to have kept within the lines of propriety, should have confined himself to a discussion of the relations of dentists to one another. The assumption of the position of censor of what the profession may or may not discuss is in line with the recent attack on this journal. Who, may we ask, constituted the editor of the *Cosmos* the censor of the profession? What was said at the meeting of the New York Odontological Society was the merest ripple upon the deep-running stream of opposition to such presumption, and the sharp manner in which the *Cosmos* tried to quell the uprising has not tended to quiet matters.

In conclusion, we desire to say that the position of this journal is one of entire independence, and *our business* the interest of the profession. We therefore insist on the right, not only for ourselves, but of each and every worker in the profession, to free expression on all matters relating to general interests, whether it be the relation of dentists to one another or to the dealer or manufacturer who furnishes our supplies, and consider him an autocrat indeed who attempts to abridge our right to free speech, by pronouncing impersonal criticism impertinent intermeddling.

